# The Application of Scrotoscope-Assisted Minimally Invasive Excision for Epididymal Mass: An Initial Report

**DOI:** 10.3389/fsurg.2022.804803

**Published:** 2022-02-24

**Authors:** Chuying Qin, Jinrui Yang, Ruochen Zhang, Yaojin Yang, Wanghai Cai, Tao Li, Qingguo Zhu, Liefu Ye, Yunliang Gao, Yongbao Wei

**Affiliations:** ^1^Department of Urology, The Second Xiangya Hospital of Central South University, Changsha, China; ^2^Shengli Clinical Medical College of Fujian Medical University, Fuzhou, China; ^3^Department of Urology, Fujian Provincial Hospital, Fuzhou, China

**Keywords:** epididymal mass, minimally invasive, open excision, scrotoscope, scrotal disease

## Abstract

**Background:**

To compare the middle-term efficacy and safety results between scrotoscope-assisted (SA) minimally invasive excision and traditional open excision (OE) for the treatment of epididymal mass.

**Methods:**

A total of 253 males with surgery excision of epididymal mass from 2012 to 2018 were included in this retrospective study. Patients were divided into two groups: the traditional OE group and the SA group. Patient demographics and intraoperative and postoperative outcomes were obtained and compared between these two groups.

**Results:**

About 174 patients (68.8%) underwent SA, and the other 79 (31.2%) underwent OE. Demographic data were similar between the two groups. Compared with OE surgery, SA could significantly shorten the operating time (19.4 ± 4.1 vs. 53.8 ± 12.9 min), reduce blood loss (5.3 ± 1.5 vs. 21.3 ± 5.6 ml), and downsize the operative incision (1.5 ± 0.3 vs. 4.5 ± 0.8 cm). Additionally, postoperative complications were significantly less occurred in the SA group than those in OE (15.5% vs. 21.5%), in particular scrotal hematoma (1.7% vs. 12.7%) and incision discomfort (2.8% vs. 6.3%). Patients in the SA group had a significantly higher overall satisfaction score (94.8 ± 3.7 vs. 91.7 ± 4.9) and a significantly shorter length of hospital stay (4.1 ± 0.9 vs. 5.0 ± 1.5 days) than those in the OE group. No postoperative testicular atrophy occurred in the SA group.

**Conclusion:**

SA is emerging as a novel and effective option with promising perspectives for epididymal mass therapy.

## Introduction

Epididymal mass is recognized as a common disorder in the male population but still seems to be a diagnostic and therapeutic dilemma. The most prominent types of epididymal mass are mass-forming epididymitis (1, 2), epididymal cyst (3), epididymal sperm granuloma (4, 5), epididymal tuberculosis, and so on. Primary tumors of the epididymis origin are rarely occurred, accounting for about 2.5% of male genital tumors (6) and at most 0.03% of all male cancers (7). Adenomatoid tumor is the most common type of epididymal tumors. Epididymal masses are almost always benign without specific treatments. However, patients are admitted to the hospital due to different degrees of scrotal symptoms such as scrotal distention, chronic pain, or tenderness. When an epididymal mass is a suspected malignant tumor or does not benefit from conservative treatments, surgical interventions appear to be considered (8–10).

For the surgical treatment of epididymal mass, the traditional open excision (OE) of mass is one of the main choices. However, OE is a non-minimal invasive treatment for scrotal diseases and brings relatively more postoperative discomfort and complications (hematomas, infection, etc.) (11, 12). Firstly reported by Shafik and Gerris et al. (13, 14), the scrotoscopy has been found to be a minimally invasive and less complicated operation for the diagnosis and treatment of scrotal diseases. As described previously, we have successfully applied scrotoscopy to manage different scrotal diseases including epididymal cyst, adult testicular hydrocele, testicular rupture, testicular torsion, and all achieved satisfactory results (15–19). In order to further improve surgical outcomes, this study was carried out for the evaluation of the feasibility and efficacy of the scrotoscope-assisted (SA) excision of epididymal masses.

## Materials and Methods

### Study Population

A retrospective analysis was performed at the Second Xiangya Hospital and the Fujian Provincial Hospital from January 2002 to 2018. Study approval was obtained from the Ethics Committee of Fujian Provincial Hospital (K-2019-10-03). Epididymal mass was diagnosed by scrotum ultrasound. The patient was included under the following conditions: aged 18 to 60 years old, diagnosed with epididymal mass, and failed conservative treatments with or without obvious symptoms. All patients underwent a clinical assessment including vital signs, ECG, and laboratory examinations (hemostasis parameters, routine hematology, liver and renal function, etc.). We conducted C-reactive protein, purified protein derivative and chest radiography, urinary ultrasound or KUB + IVP, CT, and/or scrotum MRI to exclude epididymis tuberculosis or malignant tumor. Cases with severe cardiopulmonary diseases or coagulation disorders were also excluded. All patients were fully informed about the advantages and disadvantages of each surgical approach, and the patients had their own right to determine which one to choose.

### Main Surgical Procedures

All patients received general anesthesia and operated in bladder lithotomy position with routine skin preparation. Disposable plastic incise drape was pasted in the operation area after sterilization.

**Figure 1:**
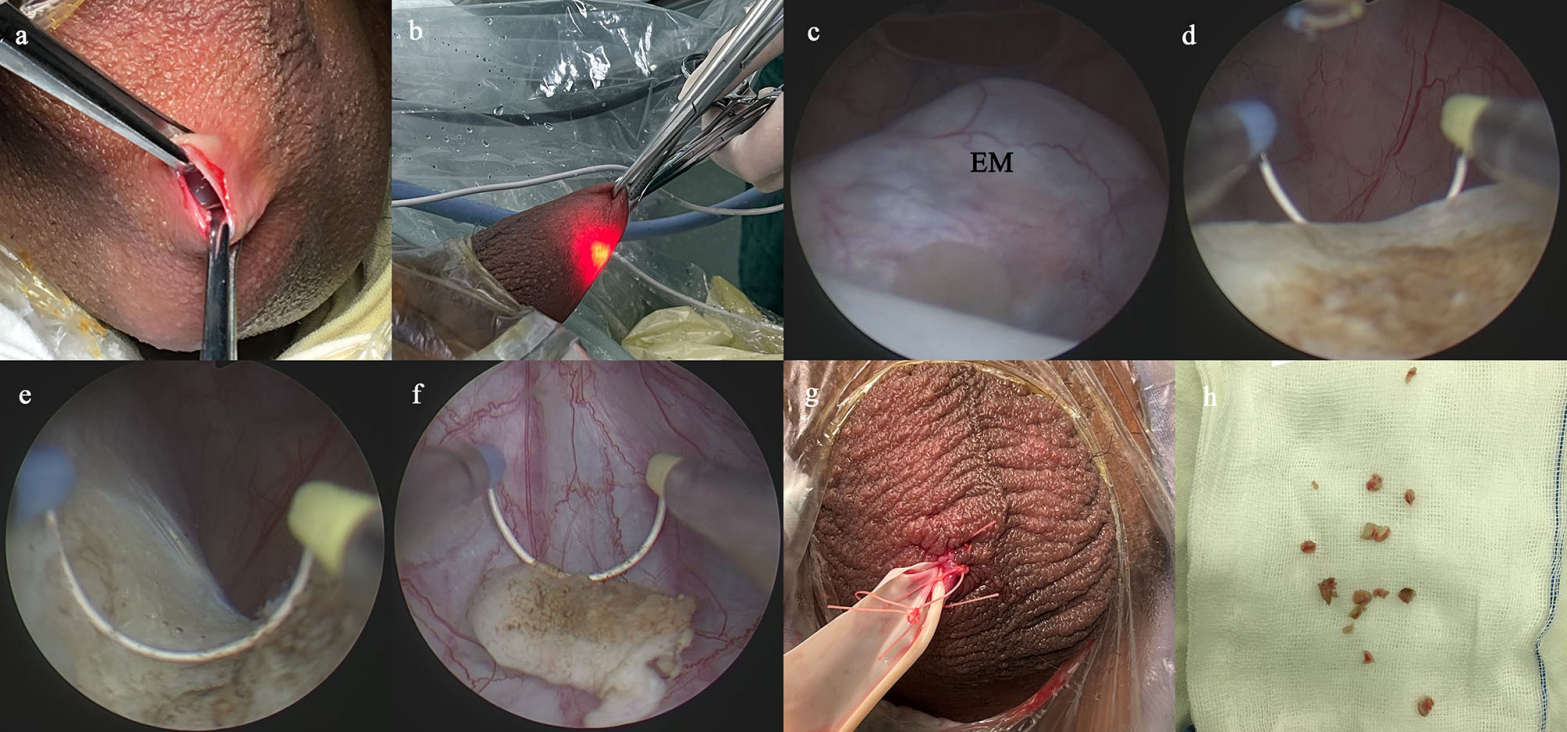
Main surgical procedures of scrotoscope-assisted epididymal mass (EM) excision. **(a)** A 1.0-cm incision is established on the affected side of the scrotum, then two Allis forceps hold the whole scrotal wall. **(b)** The scrotoscope is put into the tunica sac, and the scrotal contents were inspected sequentially. **(c)** The location, appearance, size, and margin of the EM are mainly observed. **(d)** Electrosurgical excision of the EM by plasma electroresection is performed. **(e)** The wound gets electrocoagulation to stop bleeding. **(f)** The resected fragments of mass are retrieved. **(g)** A drainage strip is placed into the scrotum. **(h)** The resected fragments of mass are sent for pathological examination.

The SA procedure was performed as previously described (15, 17, 20). Briefly, a 1.0-cm incision was established on the affected side of the scrotum, and then dissected through the scrotal layer into the tunica sac (Figure 1a). Two Allis forceps were used to hold the whole scrotal wall (Figure 1b). A 10-F cystoscope was employed as scrotoscope and put into the tunica sac with continuous isotonic crystalloid solution infusion. The crystalloid solution was suspended at a height of 60–80 mm. The scrotal contents, including the testicle, epididymis, and tunica vaginalis, were inspected sequentially. Concurrently, the location, appearance, size, and margin of the epididymal mass were mainly observed. Then, electrosurgical excision of the epididymal mass was conducted by using a traditional resectoscope in a systematic fashion, taking down gradually from the caput side to cauda side and reaching deeply to the plane between the epididymis and the testicle (Figures 1c–f). Testicular injury should be carefully avoided. After excision, the wound got electrocoagulation to stop bleeding. The resected fragments of mass were retrieved using the Ellik evacuator and sent for pathological examination (Figure 1h). A scrotoscopy was re-performed to examine the scrotal contents to exclude any active bleeding or neglected lesion. The incision was sutured with absorbable stitches. A drainage strip was placed into the scrotum and removed after 24–48 h (Figure 1g).

For those patients treated by the OE procedure, an about 4-cm anterior scrotal incision was made in the ill side. The epididymis and the testicle were taken out of the incision, and the epididymal mass was inspected and excised. Like SA, the incision was then closed, and a drainage strip was also used and removed within 24–48 h.

### Outcome Measurements

A descriptive analysis of patient demographics was performed, including age, time since onset, follow-up period, and the characteristics of epididymal mass (location and size). The mass size was defined as the greatest diameter recorded on ultrasound recording. Surgical details mainly included intraoperative [operating time, incision size, and blood loss = (gauze weight after wiping all blood loss – dry gauze weight) g/1.05 g/ml)] and postoperative (frequency of dressing changes, complications, and hospital stay) results. Postoperative complications were recorded in detail and graded by Clavien–Dindo system, which could be applied as a widely used tool to assess and report postoperative complications in general surgery (21, 22). All patients completed at least one follow-up visit within 6 months after surgery. During the follow-up period, the patients completed the survey of the overall satisfaction of surgical treatment (ranged from 0 to 100 score) (23).

### Statistical Analysis

Data were expressed respectively in terms of mean ± SD for normally distributed continuous values and as median with range for non-normally distributed data, whereas discrete ones were reported using percentages. We compared continuous variables with *t*-tests and used a *p*-value of less than 0.05 as a cutoff for statistical significance. A multivariable logistic analysis with a likelihood ratio test was performed to identify predictors of satisfaction. All data entry and analysis were carried out in the SPSS 24.0 statistical analysis software (SPSS, Chicago, IL, USA).

## Results

### General Information

A total of 253 patients with epididymal masses were enrolled in this retrospective study, 174 underwent SA and 79 underwent OE. The mean age was 47 ± 12.8 (22-80) years in SA and 48 ± 14.9 (22-80) years in OE, respectively. The mean follow-up time was 20.8 ± 8.2 months in SA vs. 19.4 ± 8.6 months in OE. Table 1 presents a summary of patients' characteristics. Two groups showed no significant differences in terms of age, time since onset, follow-up period, mass size (maximal diameter) on ultrasound, and the side of treatment.

**Table 1:** Demographic characteristics between groups.

	**SA** **(***n*** = 174)**	**OE** **(***n*** = 79)**	***P*** **-value**
Age (year)	47.9 ± 12.8	48.0 ± 14.9	0.962
Duration of disease (year)	2.5 ± 0.6	2.5 ± 0.6	0.403
Maximum diameter (cm)	3.2 ± 1.1	3.1 ± 1.1	0.608
Follow-up time (year)	20.8 ± 8.3	19.4 ± 8.6	0.219
Mass side			
Left	80	40	0.492
Right	94	39	
Location			
Caput	36	15	0.937
Corpus	53	26	
Cauda	42	17	
Diffuse	43	21	

*OE, open excision; SA, scrotoscope-assisted excision*.

### Intraoperative Data

All patients underwent surgery successfully. The mean operating time in SA was significantly shorter than OE (19.4 ± 4.1 vs. 53.8 ± 12.9 min). The blood loss in SA was significantly less than OE (5.3 ± 1.5 vs. 21.3 ± 5.6 ml). The mean incision size in SA was significantly shorter than that in OE (1.5 ± 0.3 vs. 4.5 ± 0.8 cm).

### Postoperative Data

**Figure 2:**
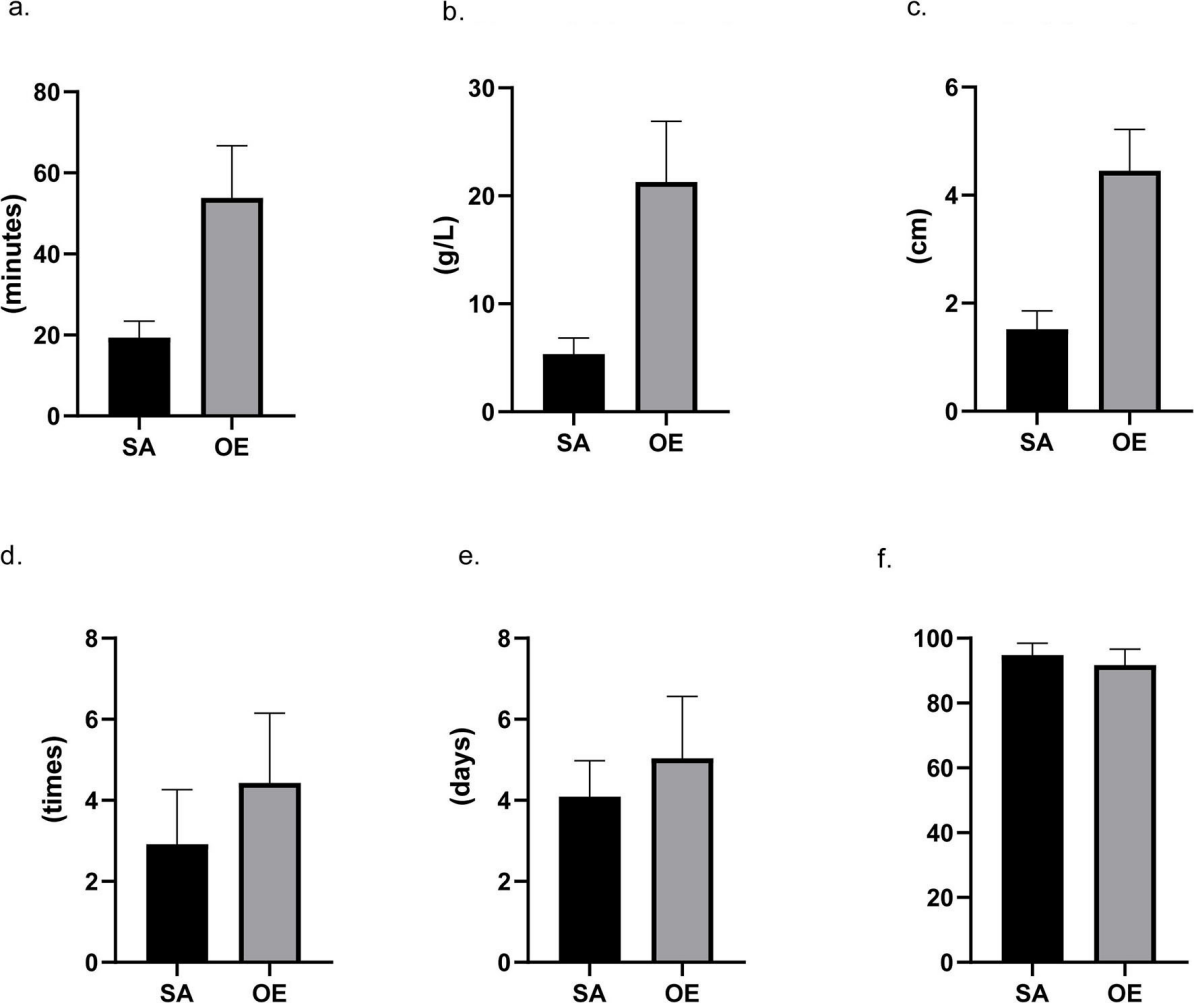
Intraoperative and postoperative data. Scrotoscope-assisted (SA) excision showed less operating time, less blood loss, shorter length of incision, and less frequency of dressing changes. SA presents no significant advantage in the number of hospital stays. Furthermore, compared to open excision, SA also leads to a higher score in satisfaction (all *p*-value < 0.05). **(a)** Operating time. **(b)** Hemoglobin reduction. **(c)** Incision size. **(d)** Frequency of dressing changes. **(e)** Hospital stay. **(f)** Satisfaction score.

Patients in the SA group had a significantly less frequency of dressing changes (2.9 ± 1.3 vs. 4.4 ± 1.7 times) and a significantly shorter length of postoperative hospital stay (4.1 ± 0.9 vs. 5.0 ± 1.5 days) when compared with the OE group (Figure 2). A significantly higher overall satisfaction score was found in the SA group rather than in the OE group (94.8 ± 3.7 vs. 91.7 ± 4.9). Moreover, the multivariable logistic analysis showed satisfaction was significantly associated with age, surgical approach, perioperative factors (operation time, blood loss, incision size, and frequency of dressing changes), and postoperative complications (all *p* < 0.05).

No significant difference was reported for the total incidence of complications and complication Grades, which is 27 (15.5%) in the SA group and 17 (21.5%) in the OE group (Grades I–III) (Table 2). Among them, almost equal incidence of postoperative recurrence was observed, which were 5 (2.9%) in SA and 2 (2.5%) cases in OE. The number of scrotal edema that occurred in SA were 19 (10.9%) cases, whereas in OE was 0 (0%). However, the incidence of scrotal hematoma in SA was less than OE, which were 3 (1.7%) vs. 10 (12.7%) cases. Fewer cases suffered incision discomfort in SA, which were 5 (2.8%) cases in SA vs. 5 (6.3%) cases in OE. No testicular atrophy (0%) occurred in SA but 1 (1.3%) case in OE. No testicular, spermatic cord injury or secondary hydrocele occurred. Except for cases with slight scrotal discomfort after operation, all the other patients' preoperative symptoms were significantly relieved.

**Table 2:** Complications and classification between groups.

		**SA (%)**	**OE (%)**	***P*** **-value**
Total complications	No	147 (84.5)	62 (78.5)	0.243
	Yes	27 (15.5)	17 (21.5)	
Complication classification	I	22 (12.6)	12 (15.2)	0.099
	II	5 (2.9)	4 (5.1)	
	III	0 (0)	1 (1.3)	
Relief of symptoms	Complete	155 (89.1)	66 (83.5)	0.134
	Partial	18 (10.3)	10 (12.7)	
	None	1 (0.6)	3 (3.8)	
Recurrence	No	169 (97.1)	77 (97.5)	0.878
	Yes	5 (2.9)	2 (2.5)	
Scrotal edema	No	155 (89.1)	79 (100)	0.009
	Slight	15 (8.6)	0 (0)	
	Severe	4 (2.3)	0 (0)	
Scrotal hematoma	No	171 (98.3)	69 (87.3)	0.000
	Yes	3 (1.7)	10 (12.7)	
Incision discomfort	No	169 (97.1)	74 (93.7)	0.191
	Yes	5 (2.9)	5 (6.3)	
Testicular atrophy	No	174 (100)	78 (98.7)	0.137
	Yes	0 (0)	1 (1.3)	

*OE, open excision; SA, scrotoscope-assisted excision*.

## Discussion

Our study firstly highlighted the feasibility of scrotoscopy in the treatment of epididymal masses. As shown by our results, SA demonstrated significant superiority over traditional OE for the treatment of epididymal mass.

Traditional open resection of epididymal mass or open-partial epididymectomy was most commonly chosen for epididymal mass surgical treatment in the patients who failed medical treatment with/without obvious symptoms, or suspected malignant tumor (8–10, 24). However, open surgery had a relatively larger trauma and higher postoperative morbidity in particular hematoma and infection, which was not conducive to the rapid recovery of patients (25–27). It is a necessity to develop a minimally invasive approach in surgical treatment of scrotal diseases. The scrotoscopy has been proven to be a minimally invasive and less complicated operation for the diagnosis and treatment of intrascrotal diseases in the past decades (13, 14). We had also applied scrotoscopy for the management of several scrotal diseases and achieved favorable results (16–18, 20, 28, 29). Compared with the traditional open approach, the scrotoscope-aided one has obvious advantages such as a shorter operation time, a smaller incision, and a faster recovery after surgery of fast, minimally invasive, rapid recovery, and fewer complications (15).

Consistently, we applied scrotoscopy for the treatment of epididymal masses and achieved superior results over the traditional approach. In this study, SA showed significantly less operating time, less blood loss, and a shorter length of incision than that in OE. Possible reasons for these results are given below. Mean operating time in SA was less than OE because more time was needed to achieve hemostasis in OE. The endoscope can easily insert into the cavity of the tunica vaginalis and can easily observe the space-limited cavity, so the incision does not need to be as long as the OE. With the application of electric cutting technology, no obvious bleeding was observed during the SA process. These results support that scrotoscope could serve as a feasible and efficient tool for the treatment of epididymal mass.

Re-emphasizing the safety for scrotoscope to treat epididymal mass, the SA group showed fewer postoperative complications and a faster recovery when compared with the OE group. According to the Clavien–Dindo grading system, all the postoperative complications in two groups were Grades I–III. One Grade III case was reported in the OE group, but none in the SA group. Scrotal edema was the most common complication in SA; this may be related to the damage of epididymis or extra fluid infiltration into the interlayer of the scrotal wall through the incision. Generally, avoiding wall sheath damage and reducing perfusion pressure and time can effectively avoid edema (15, 20). Additionally, one concern should be mentioned that high pressure resulting from hydrodistension may cause further damage to the ipsilateral testicle. However, this pressure-related damage to the testicle was not notable in our study, possibly covered by short-term scrotal edema. Long-term follow-up, such as semen analysis, may be necessary. The operators should control the hydraulic pressure within a proper range. Based on our own experience, a maintained 60–80-cm hydraulic pressure is preferred. Scrotal hematoma, however, was the most common complication in OE rather than in SA, possibly due to the relatively larger surgical trauma in an open procedure. For SA patients, the mass could be minimally excised under scrotoscope, and the bleeding site could be electrocoagulated without extruding the testicle from the scrotum, minimizing the risk of bleeding. Of note, no case of testicular atrophy occurred in the SA group but one in the OE group, potentially due to no extrusion of the testicle and the spermatic cord from the scrotal incision. Besides, SA was performed under clear direct vision to avoid severe damage. One point should be taken into account that a chromatic aberration of the digital cameras may lead to a biased color of the testis. When performing electrosurgical excision of the epididymal mass, the operators should make sure which part belongs to the testis and figure out the plane between the epididymis and the testis. As shown in Table 2, patients with SA also lead to a significantly higher rate of symptom relief and higher score in satisfaction, suggesting that patients were highly satisfied with SA. These may be attributed to multiple advantages of SA as manifested by the logistic analysis. Patients with SA experienced less incision discomfort, less trauma, fewer dressing changes, fewer postoperative complications, and shorter hospital stay, all of which contributed to the higher satisfaction. Thus, the choice of surgical approach was a relatively controllable factor and highly related to patients' satisfaction. When it comes to postoperative recurrence, there was no significant difference between the two groups. SA had less frequency of dressing changes, probably due to the shorter incisions and better bleeding control.

However, certain limitations should be addressed in this research, including the retrospective nature, the limited size of the total population, and lack of long-term follow-up data. Furthermore, the SA and OE groups were not perfectly matched by age, size of lesion, and etiology for the operation. The estimation of blood loss in the SA group may be inaccurate, which would reduce the confidence power. Moreover, the postoperative length of stay was relatively long for such a scrotal surgery under scrotoscope, possibly due to previous local medical insurance policy. In addition, patients with SA preferred to stay longer due to the concern of this uncommon surgical procedure to the public. Future studies with larger sample sizes should be conducted to further validate its diagnostic and therapeutic value.

In conclusion, our present study confirmed that SA was a safe and effective minimally invasive therapeutic option for epididymal mass. It has the advantages of small incision, rapid recovery, and low risk of complications. It is worthy of further clinical application.

## Data Availability Statement

The original contributions presented in the study are included in the article/supplementary material, further inquiries can be directed to the corresponding author.

## Ethics Statement

The studies involving human participants were reviewed and approved by K-2019-10-03/Ethics Committee of Fujian Provincial Hospital. Written informed consent for participation was not required for this study in accordance with the national legislation and the institutional requirements. Written informed consent was obtained from the individual(s) for the publication of any potentially identifiable images or data included in this article.

## Author Contributions

YW and YG conceived of the presented idea and encouraged JY, RZ, YY, WC, TL, QZ, and LY to collect data and supervised the findings of this work. YW developed the theory and performed the computations. CQ verified the analytical methods and wrote the manuscript with support from YG. All authors discussed the results and contributed to the final manuscript.

## Funding

Support was provided by the Startup Fund for scientific research, the Fujian Medical University (Grant number: 2019QH1154), and the Natural Science Foundation of Fujian Province (Grant number: 2021J01359).

## Conflict of Interest

The authors declare that the research was conducted in the absence of any commercial or financial relationships that could be construed as a potential conflict of interest.

## Publisher's Note

All claims expressed in this article are solely those of the authors and do not necessarily represent those of their affiliated organizations, or those of the publisher, the editors and the reviewers. Any product that may be evaluated in this article, or claim that may be made by its manufacturer, is not guaranteed or endorsed by the publisher.
